# Emergence of plasmid-mediated mcr genes from Gram-negative bacteria at the human-animal interface

**DOI:** 10.1186/s13099-020-00392-3

**Published:** 2020-11-20

**Authors:** Humera Javed, Sidrah Saleem, Aizza Zafar, Aamir Ghafoor, Ahmad Bin Shahzad, Hasan Ejaz, Kashaf Junaid, Shah Jahan

**Affiliations:** 1 Department of Microbiology, University of Health Sciences, Lahore, Pakistan; 2 Department of Microbiology, The Children’s Hospital & The Institute of Child Health, Lahore, Pakistan; 3 University Diagnostic Laboratory, University of Veterinary and Animal Sciences, Lahore, Pakistan; 4 Department of Clinical Laboratory Sciences, College of Applied Medical Sciences, Jouf University, Al Jouf, Saudi Arabia; 5 Department of Immunology, University of Health Sciences, Khayaban-e-Jamia Punjab, 54600 Lahore, Pakistan

**Keywords:** Antibacterial profile, Antimicrobial resistance, Colistin resistance, *mcr* genes, Mobile colistin resistance, Plasmid-mediated resistance

## Abstract

**Background:**

The global emergence of plasmid-mediated colistin resistance (Col-R) conferred by *mcr* genes in gram-negative rods (GNRs) has jeopardized the last treatment option for multidrug-resistant bacterial infections in humans. This study aimed to assess the emergence of *mcr* gene-mediated Col-R in GNRs isolated from humans and animals in Pakistan.

**Methods:**

Animal and clinical specimens collected from various sources were prospectively analysed using standard microbiological procedures. Pathogens were identified using the API 20E and API 20NE systems (bioMerieux). Minimum inhibitory concentration (MIC) against colistin was determined using the MIC detection methods, and multiplex polymerase chain reaction (PCR) was used to amplify the *mcr-1* to *mcr-5* genes.

**Results:**

We isolated 126 (88.1%) animal and 17 (11.9%) human Col-R phenotypes, among which there was a significant association (P < 0.01) of *Escherichia coli* and *Proteus mirabilis* with animals and of *Acinetobacter baumannii* with humans. Animal strains exhibited statistically significant (P < 0.05) resistance to co-trimoxazole, chloramphenicol, and moxifloxacin, and the human pathogens exhibited statistically significant (P < 0.05) antibiotic resistance to cephalosporins, carbapenems, and piperacillin-tazobactam. For Col-R strains, MIC_50_ values were > 6 µg/mL and > 12 µg/mL for human and animal isolates, respectively. *mcr* genes were detected in 110 (76.9%) bacterial strains, of which 108 (98.2%) were *mcr-1* and 2 (1.8%) were *mcr-2*.

**Conclusions:**

The detection of a considerable number of *mcr-1* and *mcr-2* genes in animals is worrisome, as they are now being detected in clinical pathogens. The acquisition of *mcr* genes by colistin-susceptible bacteria could leave us in a post-antibiotic era.

## Background

Continuously emerging antibiotic resistance poses a serious survival challenge to humankind and is leading us into a post-antibiotic era. The emergence of superbugs carrying extended-spectrum beta-lactamases (ESBLs), AmpC beta-lactamases, and metallo-beta-lactamases has reduced therapeutic choices [1]. Colistin is a cyclopeptide antibiotic prescribed as a last resort for the treatment of extensively drug-resistant (XDR) bacteria [2]. This drug was discovered more than seven decades ago and was first introduced in the 1960s for clinical use. It was replaced with other antibiotics in the 1970s because of its nephrotoxic and neurotoxic effects. Since then, it has been introduced into veterinary medicine [3]. Colistin attracted renewed attention and was reintroduced as an emergency solution in the 1990s in response to the escalating prevalence of XDR bacteria [4].

The situation became alarming because of the emergence of mobile colistin resistance (*mcr*) genes, initially in China, in animals and humans [5]. To date, more than 40 countries have reported *mcr* variants (*mcr-1* to *mcr-9*) from five different continents across the globe, indicating the epidemicity of the *mcr* gene [6]. The *mcr* genes have been reported in seven Asian and nine European countries, and they were recently identified in Pakistan, Iran, Italy, Finland, America, South Africa, and some other territories [7–10]. The plasmid-borne *mcr* gene has been found in several enterobacteria, including *Escherichia coli*, *Salmonella*, *Aeromonas*, *Enterobacter cloacae, Klebsiella pneumoniae, Escherichia fergusonii, Kluyvera ascorbata, Citrobacter braakii, Cronobacter sakazakii, Klebsiella aerogenes*, and, most recently, *Raoultella ornithinolytica* [6].

Poultry and livestock, including chickens, ducks, pigeons, geese, pigs, and cattle, have been reported to be reservoir hosts for mcr-harbouring bacterial strains [11]. Of particular note, the animal-to-human transmission of *mcr-1* colistin resistance (Col-R) has already been established in China, Thailand, Laos, and Denmark, which has raised a serious concern about its possible global dissemination [5, 12, 13]. In addition to their isolation from animals and humans, *mcr* genes have also been reported in bacteria from sewage, seawater, fresh food products, and seafood [14].

The extensive veterinary use of colistin and the increasing reports of Col-R in food animal strains of enterobacteria are indeed a matter of concern. The large-scale subclinical use of colistin for prophylaxis and growth promotion in livestock is a major cause of resistance [6]. The expansion of Col-R to various countries has led us to evaluate the magnitude of this drug resistance phenomenon in Pakistan. In this study, we aimed to assess the plasmid-mediated Col-R conferred by *mcr* genes among gram-negative rods (GNRs) isolated from humans and animals. The resistance spectrum of the Col-R and multidrug-resistant GNRs to a variety of antibiotics was elucidated to identify possible therapeutic regimens for combating these superbugs.

## Methods

### Study design and setting

The study was conducted prospectively over 18 months according to the ethical principles provided by the World Medical Association (WMA) Declaration of Helsinki. The study collaborated with and received ethics approval from the University of Health Sciences and the Children’s Hospital and the Institute of Child Health, Lahore, Pakistan.

### Sample collection

A total of 38,500 human clinical specimens were randomly collected from the tertiary care public and private hospitals in Lahore, which treat patients from all over the Punjab province (population of approximately 120 million). The specimens collected from the clinical settings included blood, cerebrospinal fluid, swabs, tracheal secretions, urine, and faeces. Animal meat, chicken faecal, and respiratory secretion specimens (n = 630) were collected from different retail shops. We also collaborated with the University Diagnostic Laboratory (UDL), University of Veterinary and Animal Sciences, Lahore, Pakistan, to collect bacterial strains. UDL analyses animal pathological samples from all over the province.

### Microbiological identification

The clinical specimens from sterile sites were processed for culture using blood, chocolate, and MacConkey’s culture media [15]. Urine samples were cultured on cysteine lactose electrolyte deficient (CLED) media, while xylose lysine deoxycholate (XLD) agar was used to culture the faecal specimens. The samples collected from the animal sources were processed on XLD and MacConkey’s agar. The bacterial cultures were identified using standard microbiological techniques, including Gram staining, oxidase production, and the API 20E and API 20NE systems (bioMerieux, France). Only GNRs resistant to colistin were included and processed further in our study.

### Minimum inhibitory concentrations (MICs) against colistin

The GNRs recovered from both human and animal sources were tested for Col-R using E-test strips (Liofilchem, Italy), and the selected strains were confirmed with the SensiTest™ Colistin (Liofilchem, Italy). Only the Col-R strains were included in the study for further processing. The minimum inhibitory concentrations (MICs) were determined using an MIC epidemiological cut-off value (ECV) ≤ 2 µg/mL for the wild-type (WT) strains and ≥ 4 µg/mL for the non-wild-type (NWT) strains [16]. In the culture media and disc diffusion techniques, the ATCC 25922 (colistin-sensitive *E. coli*) and ATCC 25933 (colistin-resistant *Proteus mirabilis*) strains were used for quality control (QC).

### Disc diffusion antibiotic testing

The Col-R phenotypes were assessed further to determine the association with drug resistance in the human and animal strains [16]. Antibacterial drug resistance against 16 other drugs that belong to several classes of antibiotics was tested using the disc diffusion method [16, 17]. The discs primarily used included aminoglycosides, cephalosporins, fluoroquinolones, carbapenems, and beta-lactam combinations.

#### Molecular detection of ***mcr*** genes and data analysis

The bacterial DNA from the freshly cultured GNR strains was thermally extracted by emulsifying 2–3 colonies in 200 µL of Tris EDTA (TE) buffer and boiling for 10 min [18]. Previously described *mcr1* to *mcr5* primers were used in the multiplex polymerase chain reaction (PCR) [19]. The amplification was performed on a thermal cycler (Biorad, T1000) using 12.5 µL Dream Taq master mix (Thermo Fisher Scientific, USA), 0.5 µL each of the 10 forward and reverse primers (10 µM), 5.5 µL nuclease-free water, and 2 µL of bacterial DNA in a final reaction mixture of 25 µL. The amplification procedure comprised an initial denaturation step at 94 °C for 15 min followed by 25 cycles at 94 °C for 30 s, 58 °C for 90 s, 72 °C for 60 s, and a final extension at 72 °C for 10 min [19]. The amplified *mcr* gene products were loaded on a horizontal agarose gel electrophoresis apparatus using 6 × loading dye and SYBR^™^ Safe DNA gel stain (Invitrogen). A 100 bp ladder was included with each electrophoresis run, and the gene bands were visualized with a gel documentation system (EZ Imager Bio-Rad). GraphPad Prism 7 and SPSS 23 were used for the statistical analysis. Chi-square tests were used to examine the association of antimicrobial drug-resistant microorganisms with the animal and human sources, and a significance threshold was set at P < 0.05.

## Results

### Association of Col-R in human and animal isolates

A total of 5,893 (15.3%) gram-negative rods from 38,500 human clinical specimens and 630 (49%) from 1,285 animal samples were identified. The distribution of colistin-sensitive (Col-S) and Col-R gram-negative strains isolated from human and animal sources is given in Table 1. We identified 143 gram-negative Col-R phenotypes from the human and animal isolates, of which 126 (88.1%) were isolated from animal and 17 (11.9%) from human sources. Among the animal isolates, 91 (72.2%) *E. coli* and 20 (15.9%) *P. mirabilis* strains were significantly associated (P < 0.01) with the animal sources, while 3 (17.6%) *Acinetobacter baumannii* strains were significantly associated (P < 0.01) with the human sources. There was no significant association of *K. pneumoniae* and *Pseudomonas aeruginosa* with any source (Table 2).

**Table 1:** Distribution of colistin-sensitive (Col-S) and colistin-resistant (Col-R) gram-negative strains isolated from human and animal sources

Organisms	Animal isolates (n = 1285)			Human isolates (n = 5893)		
	**Total**	**Col-S**	**Col-R**	**Total**	**Col-S**	**Col-R**
*E. coli*	658	567 (86.2%)	91 (13.8%)	2219	2216 (99.9%)	3 (0.1%)
*Klebsiella* species	231	219 (94.8%)	12 (5.2%)	1592	1591 (99.9%)	1 (0.1%)
*Pseudomonas* species	81	78 (96.3%)	3 (3.7%)	995	995 (100%)	0 (0%)
*Citrobacter* species	89	89 (100%)	0 (0%)	351	351 (100%)	0 (0%)
*Acinetobacter* species	0	0 (0%)	0 (0%)	318	315 (99.1%)	3 (0.9%)
*Enterobacter* species	85	85 (100%)	0 (0%)	260	260 (100%)	0 (0%)
*Proteus* species	20	0 (0%)	20 (100%)	10	0 (0%)	10 (100%)
*Salmonella* species	109	109 (100%)	0 (0%)	85	85 (100%)	0 (0%)
*Sphingomonas paucimobilis*	0	0 (0%)	0 (0%)	25	25 (100%)	0 (0%)
*Chryseomonas luteola*	4	4 (100%)	0 (0%)	19	19 (100%)	0 (0%)
*Pantoea* species	8	8 (100%)	0 (0%)	19	19 (100%)	0 (0%)

**Table 2:** Association of colistin resistance (Col-R) in bacteria isolated from human and animal sources (n = 143)

Organism	Col-R animal source n (%)	Col-R human source n (%)	P-value
	126 (88.1)	17 (11.9)	
*E. coli*	91 (72.2)	3 (17.6)	< 0.01
*K. pneumoniae*	12 (9.5)	1 (5.9)	0.62
*P. aeruginosa*	3 (2.4)	0 (0)	0.52
*A. baumannii*	0 (0)	3 (17.6)	< 0.01
*P. mirabilis*	20 (15.9)	10 (58.8)	< 0.01

### Distribution of Col-R strains from different specimens

**Fig. 1:**
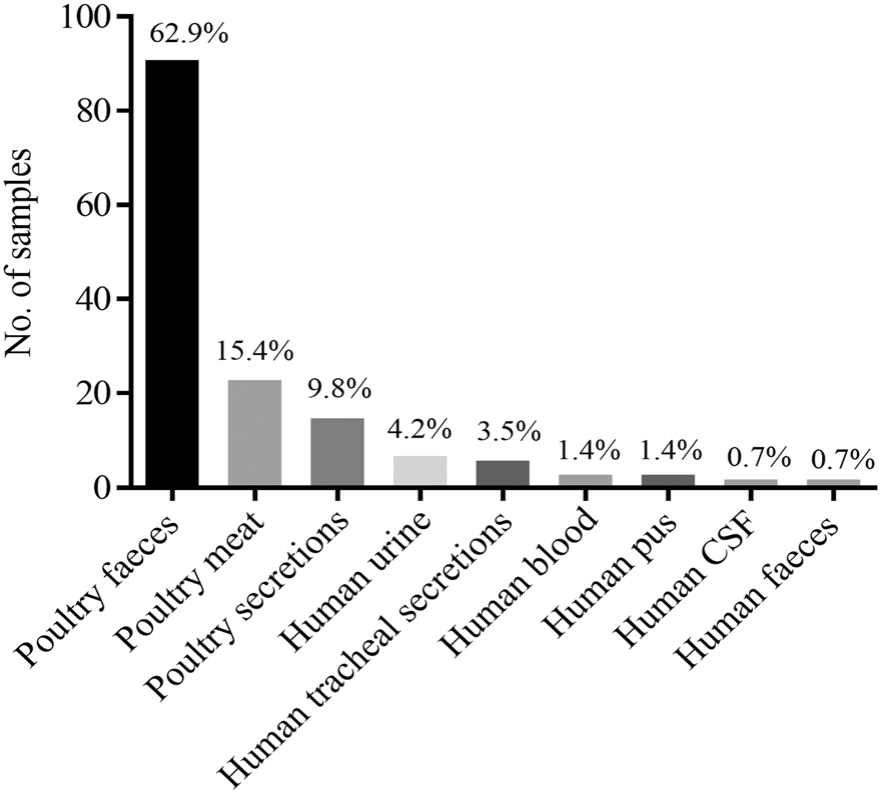
Distribution of colistin resistance (Col-R) among bacterial strains isolated from different specimens (n = 143)

**Fig. 2:**
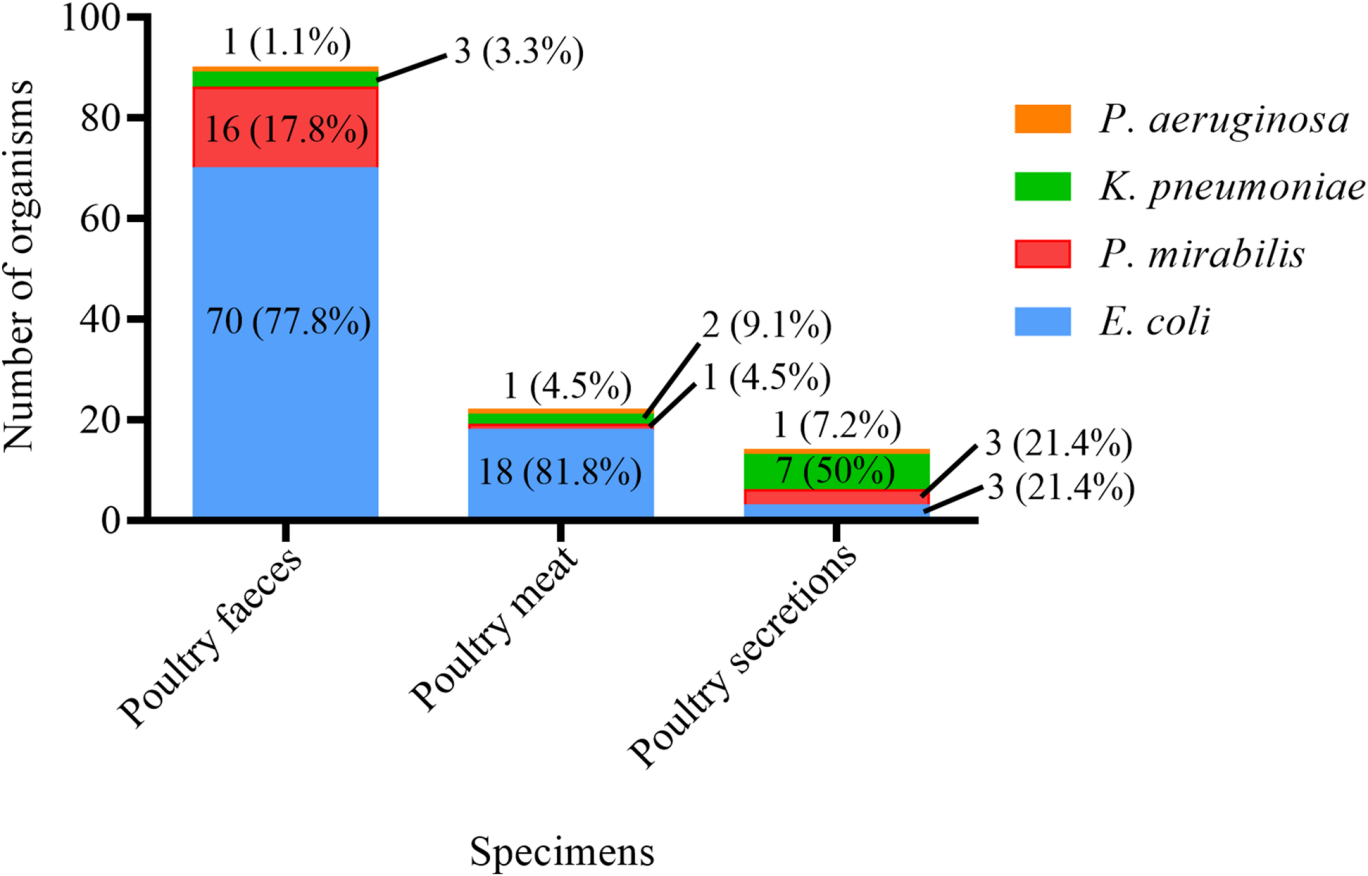
Distribution of bacterial strains in animal sources (n = 126)

The Col-R bacterial strains were predominantly found in different poultry specimens, including 90 (62.9%) in faecal, 22 (15.4%) in meat, and 14 (9.8%) in secretion samples. From the human sources, 6 (4.2%) Col-R strains were found in urine, and 5 (3.5%) were found in tracheal secretions, while the rest of the strains were found in the other human specimens (Fig. 1). The predominant source was poultry faeces, with 70 samples (77.8%) containing *E. coli* and 16 (17.8%) containing *P. mirabilis*, followed by poultry meat, with 18 samples (81.1%) containing *E. coli*. The frequencies of bacterial isolates from each animal source are shown in Fig. 2.

### Drug-resistance spectrum against various antibiotics

For the animal strains, there was a statistically significant association of drug resistance to co-trimoxazole (80.2% vs. 47.1%; P = 0.01), chloramphenicol (73% vs. 52.9%; P = 0.05), and moxifloxacin (71.4% vs. 41.2%; P = 0.03). Meropenem and piperacillin-tazobactam had the lowest number of animal strains resistant to them, each at 10 (7.9%), followed by 12 (9.5%) strains resistant to cefoperazone-sulbactam, 16 (12.7%) to imipenem, and 20 (15.9%) to amikacin (Table 3).

**Table 3:** Association of antibiotic resistance in animal and human bacterial strains (n = 143)

Antibiotics	Animal isolates	Human isolates	P-value
	n = 126 (%)	n = 17 (%)	
Co-trimoxazole	101 (80.2)	8 (47.1)	0.01
Chloramphenicol	92 (73)	9 (52.9)	0.05
Moxifloxacin	90 (71.4)	7 (41.2)	0.03
Levofloxacin	41 (32.5)	4 (23.5)	0.59
Ciprofloxacin	42 (33.3)	4 (23.5)	0.69
Co-amoxiclav	37 (29.4)	9 (52.9)	0.05
Cefotaxime	33 (26.2)	10 (58.8)	0.01
Cefixime	32 (25.4)	9 (52.9)	0.02
Cefuroxime	29 (23)	10 (58.8)	0.007
Ceftriaxone	28 (22.2)	11 (64.7)	0.01
Ceftazidime	25 (19.8)	10 (58.8)	< 0.01
Amikacin	20 (15.9)	4 (23.5)	0.73
Imipenem	16 (12.7)	5 (29.4)	< 0.01
Cefoperazone-sulbactam	12 (9.5)	4 (23.5)	0.22
Piperacillin-tazobactam	10 (7.9)	2 (11.8)	0.05
Meropenem	10 (7.9)	5 (29.4)	0.02

For the human pathogens, there was a statistically significant association of antibiotic resistance, primarily to the cephalosporins: ceftriaxone (64.7% vs. 22.2%; P = 0.01), cefuroxime (58.8% vs. 23%; P = 0.007), cefotaxime (58.8% vs. 26.2%; P = 0.01), ceftazidime (58.8% vs. 19.8%; P < 0.01), and cefixime (52.9% vs. 25.4%; P = 0.02). There was a statistically significant difference in resistance between the human isolates and the animal isolates to the beta-lactamase-resistant drugs, which include co-amoxiclav (52.9% vs. 29.4%; P = 0.05), meropenem (29.4% vs. 7.9%; P = 0.02), imipenem (29.4% vs. 12.7%; P < 0.01), and piperacillin-tazobactam (11.8% vs. 7.9%; P = 0.05). The lowest number of strains among the human pathogens resistant to a drug was 2 (11.8%), which was for piperacillin-tazobactam, followed by 4 (23.5%) each for cefoperazone-sulbactam, amikacin, ciprofloxacin, and levofloxacin (Table 3).

*Escherichia coli* and *K. pneumoniae* isolated from animal and human sources and *P. aeruginosa* isolated from animal sources showed more resistance to co-trimoxazole, chloramphenicol, and moxifloxacin than to the other drugs. All of the *K. pneumoniae* (human sources) and *P. aeruginosa* (animal sources) strains were also resistant to levofloxacin and ciprofloxacin. *A. baumannii* strains isolated from human sources showed resistance to all the antibacterial drugs except piperacillin-tazobactam. The detailed antimicrobial resistance profiles of the individual Col-R bacterial strains are presented in Table 4.

**Table 4:** Antimicrobial resistance profiles of individual colistin-resistant bacterial strains from animal and human sources

Antibiotics	*E. coli*		*K. pneumoniae*		*P. aeruginosa*		*A. baumannii*		*P. mirabilis*	
	Animal	Human	Animal	Human	Animal	Human	Animal	Human	Animal	Human
	(n = 91)	(n = 3)	(n =12)	(n = 1)	(n = 2)	(n = 0)	(n = 0)	(n = 3)	(n = 20)	(n = 10)
Co-trimoxazole	64 (70%)	3 (100%)	5 (42%)	1 (100%)	2 (100%)	–	–	3 (100%)	8 (40%)	10 (100%)
Chloramphenicol	64 (70%)	3 (100%)	5 (42%)	1 (100%)	2 (100%)	–	–	3 (100%)	8 (40%)	4 (40%)
Moxifloxacin	59 (65%)	2(67%)	5 (42%)	1 (100%)	2 (100%)	–	–	3 (100%)	3 (15%)	4 (40%)
Levofloxacin	21 (23%)	1 (33%)	3 (25%)	1 (100%)	2 (100%)	–	–	2 (75%)	3 (15%)	1 (10%)
Ciprofloxacin	38 (42%)	0 (0%)	2 (17%)	1 (100%)	2 (100%)	–	–	3 (100%)	5 (25%)	1 (10%)
Co-amoxiclav	39 (43%)	2 (67%)	1 (8%)	1 (100%)	0 (0%)	–	–	3 (100%)	6 (30%)	1 (10%)
Cefotaxime	35 (38%)	1 (33%)	1 (8%)	1 (100%)	0 (0%)	–	–	2 (75%)	3 (15%)	1 (10%)
Cefixime	29 (32%)	2 (67%)	3 (25%)	1 (100%)	0 (0%)	–	–	3 (100%)	6 (30%)	3 (30%)
Cefuroxime	27 (30%)	2 (67%)	0 (0%)	1 (100%)	0 (0%)	–	–	3 (100%)	7 (35%)	3 (30%)
Ceftriaxone	27 (30%)	2 (67%)	1 (8%)	1 (100%)	0 (0%)	–	–	3 (100%)	7 (35%)	3 (30%)
Ceftazidime	27 (30%)	1 (33%)	0 (0%)	1 (100%)	0 (0%)	–	–	3 (100%)	1 (5%)	3 (30%)
Amikacin	16 (18%)	1 (33%)	3 (25%)	1 (100%)	0 (0%)	–	–	2 (75%)	2 (10%)	0 (0%)
Imipenem	12 (13%)	1 (33%)	3 (25%)	1 (100%)	0 (0%)	–	–	1 (33%)	0 (0%)	2 (20%)
Cefoperazone-sulbactam	7 (8%)	1 (33%)	2 (17%)	1 (100%)	1 (50%)	–	–	2 (67%)	2 (10%)	0 (0%)
Piperacillin-tazobactam	9 (10%)	0 (0%)	0 (0%)	1 (100%)	0 (0%)	–	–	0 (0%)	2 (10%)	1 (10%)
Meropenem	9 (10%)	1 (33%)	1 (8%)	1 (100%)	0 (0%)	–	–	3 (100%)	2 (10%)	0 (0%)

#### MICs of colistin against Col-R bacterial strains

**Fig. 3:**
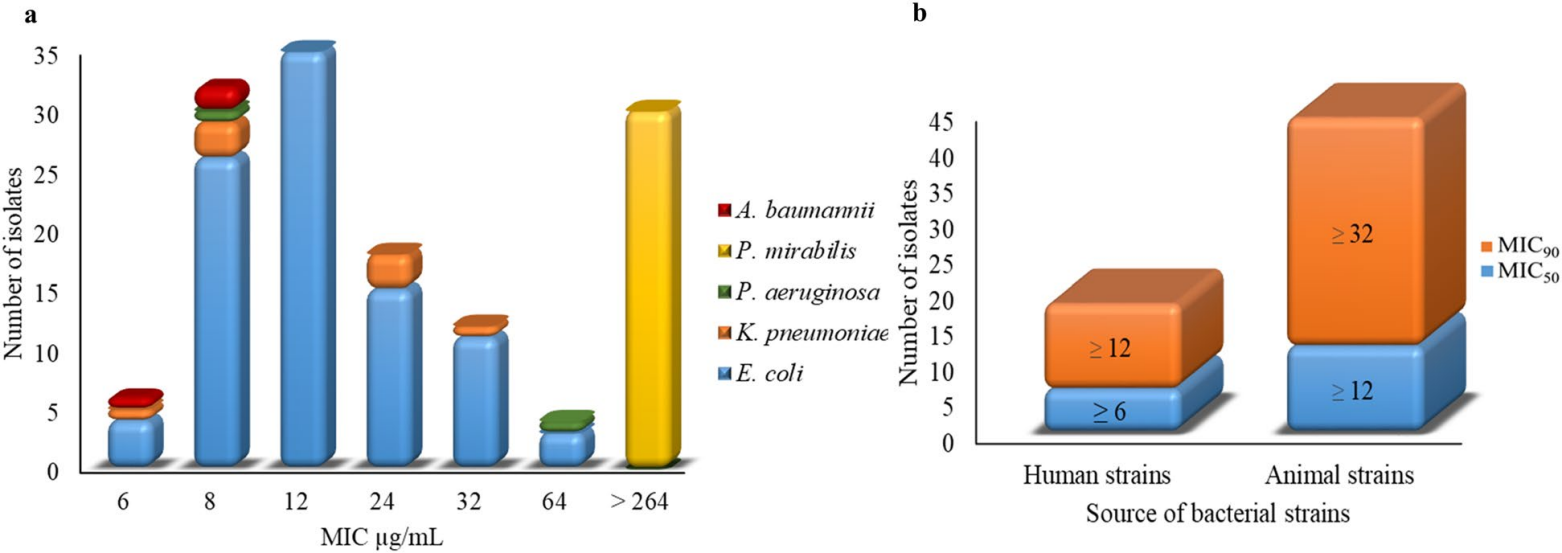
MICs of colistin against colistin-resistant (Col-R) bacterial strains isolated from animal and human samples. **a** MICs of individual bacteria isolated from animal and human sources (n = 143). MIC values for all of the *P. mirabilis* strains were > 264 µg/mL. **b** MIC_50_ and MIC_90_ of human and animal bacterial isolates (n = 113), excluding *P. mirabilis* because of its intrinsic Col-R

The MICs of colistin against bacterial strains (n = 113) from human and animal sources showed MICs of 6, 8, 12, 24, 32, and 64 µg/mL. Because of the intrinsic resistance to colistin, the MICs of all of the *P. mirabilis* strains were > 264 µg/mL. The MIC distributions of colistin against human pathogens were MIC_50_ > 6 µg/mL and MIC_90_ > 12 µg/mL. The MIC_50_ and MIC_90_ values were > 12 µg/mL and > 32 µg/mL, respectively, for the animal isolates (Fig. 3).

#### Occurrence of ***mcr*** genes in isolated bacteria

**Fig. 4:**
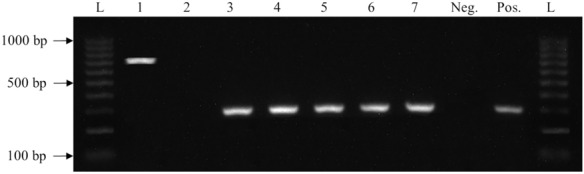
Agarose gel electrophoresis of *mcr* genes. Sample 1 shows the amplification of *mcr-2*, while specimens 3–7 show the amplified *mcr-1* gene products. A 100 bp ladder (L) was included on both sides of the gel to estimate the gene sizes. Negative (Neg.) and positive (Pos.) controls were included in each gel

All of the Col-R gram-negative bacterial strains were analysed to determine the presence of *mcr-1*, *mcr-2*, *mcr-3*, *mcr-4*, and *mcr-5*. *mcr* genes were detected in 110 (76.9%) Col-R bacterial strains from animal and human sources, of which 108 (98.2) were *mcr-1* and 2 (1.8%) were *mcr-2* (Fig. 4). For the animal pathogens, *mcr-1* was found in 90 (83.3%) *E. coli* strains, 12 (11.1%) *K. pneumoniae* strains, and 1 (0.9%) strain each of *P. aeruginosa* and *P. mirabilis*. For the human pathogens, *mcr-1* was found in 3 (2.8%) *E. coli* strains and 1 (0.9%) *K. pneumoniae* strain. Only 2 (100%) strains of *P. mirabilis* isolated from the animal sources harboured *mcr-2* (Table 5).

**Table 5:** Distribution of *mcr* genes in human and animal bacterial strains (n = 110)

Bacterial strains	*mcr-1* (n = 108; 98.2%)		*mcr-2* (n = 2; 1.8%)	
	Animal n (%)	Human n (%)	Animal n (%)	Human n (%)
	104 (96.3)	4 (3.7)	2 (100)	0 (0)
*E. coli*	90 (83.3)	3 (2.8)	0 (0)	0 (0)
*K. pneumoniae*	12 (11.1)	1 (0.9)	0 (0)	0 (0)
*P. aeruginosa*	1 (0.9)	0 (0)	0 (0)	0 (0)
*P. mirabilis*	1 (0.9)	0 (0)	2 (100)	0 (0)
*A. baumannii*	0 (0)	0 (0)	0 (0)	0 (0)

## Demographic and clinical data for the human isolates

Analysis of the demographic and clinical data of the patients infected with Col-R pathogens revealed that Col-R strains were isolated from various clinical specimens from patients of different ages and genders. The *mcr*-1 gene was detected in 3 *E. coli* (urine specimen) strains and 1 *K. pneumoniae* (blood specimen) strain, and none of the *mcr* variants were identified in any other bacterial strain of human origin (Table 6).

**Table 6:** Demographic and clinical data of patients infected with colistin-resistant pathogens (n = 17)

Gender	Age (Years)	Ward	Organism isolated	Specimen	Colistin MIC µg/mL	*mcr* gene
Male	1	Medical ICU	*K. pneumoniae*	Blood	6	*mcr*-1
Female	38	Outpatient department	*E. coli*	Urine	6	*mcr*-1
Female	3	Nephrology Ward	*E. coli*	Urine	12	*mcr*-1
Male	67	Cardiac ICU	*E. coli*	Urine	6	*mcr*-1
Female	53	Medical ICU	*A. baumannii*	Tracheal secretions	8	Not detected
Male	3	Neurosurgery ICU	*A. baumannii*	Tracheal secretions	8	Not detected
Female	61	Medical ICU	*A. baumannii*	Tracheal secretions	6	Not detected
Female	13	Outpatient department	*P. mirabilis*	Urine	≥ 264	Not detected
Male	68	Medical Ward	*P. mirabilis*	Urine	≥ 264	Not detected
Male	51	Medical Ward	*P. mirabilis*	Stool	≥ 264	Not detected
Female	8	Nephrology Ward	*P. mirabilis*	Blood	≥ 264	Not detected
Male	3	Medical ICU	*P. mirabilis*	Tracheal secretions	≥ 264	Not detected
Male	3	Ortho Ward	*P. mirabilis*	Pus	≥ 264	Not detected
Male	10	General Surgery Ward	*P. mirabilis*	Pus	≥ 264	Not detected
Male	73	Cardiac ICU	*P. mirabilis*	Tracheal secretions	≥ 264	Not detected
Female	6	Neurosurgery Ward	*P. mirabilis*	CSF	≥ 264	Not detected
Female	3	Medical Ward	*P. mirabilis*	Urine	≥ 264	Not detected

## Discussion

The global dissemination of ESBLs, AmpC, and carbapenemase-producing bacteria has narrowed the options for appropriate antibiotics to treat gram-negative bacterial infections. The effectiveness of colistin in the treatment of XDR gram-negative bacterial infections is well known. In this era of antibacterial drug resistance, a new debate has started following the emergence of Col-R bacterial strains isolated from humans and animals. These strains can disseminate the *mcr* genes to other susceptible bacterial strains [8]. Here, we found 143 GNR Col-R phenotypes, of which 88.1% were isolated from animals and 11.9% from human sources.

In our study, Col-R was predominantly observed in strains isolated from poultry faecal samples and in uropathogens isolated from hospitalized patients. Col-R is frequently observed in animal faecal strains, indicating the intestinal colonization of Col-R bacteria in these animals [20]. Col-R has been found and reported in different countries in samples from humans, animals, and the environment [5, 21–23]. The predominant Col-R strains found were *E. coli* (72.2%) and *P. mirabilis* (15.9%) from animal sources. *E. coli* (17.6%) and *P. mirabilis* (58.8%) were also the predominant strains from human sources, although the total number of Col-R strains was not high. These findings corroborate previous studies on Col-R *E. coli*, which reported rates of 8% from broiler chicken and 37.5% from pig rectal swabs [9, 24].

The treatment of infections caused by ESBL- and AmpC beta-lactamase-producing strains remains a major concern [25, 26]. The emergence of NDM-1 during the past few years has made treating these infections challenging [27]. Polymyxin B and colistin have saved patients’ lives and are considered a vital regimen for treating XDR bacterial infections [12]. Other studies have reported the use of colistin, aminoglycosides, co-trimoxazole, piperacillin-tazobactam, cefoperazone-sulbactam, and tigecycline to treat multidrug-resistant bacteria [18]. Unfortunately, Col-R isolates have emerged worldwide because of the injudicious use of colistin, particularly in veterinary medicine [5].

Here, we analysed the antibacterial activity of aminoglycosides, cephalosporins, fluoroquinolones, carbapenems, beta-lactam, and other combinations against Col-R phenotypes. We found MIC_90_ values > 12 µg/mL and > 32 µg/mL for the clinical and animal isolates, respectively. It is important to accurately determine the MICs of colistin and the detection of *mcr* genes provides valuable information to better understand the mechanism of resistance in borderline or resistant cases [19]. In our study, the animal strains were significantly resistant to co-trimoxazole, chloramphenicol, and moxifloxacin, which is consistent with the findings of a Thai study [28]. Interestingly, we noticed lower resistance to carbapenems, piperacillin-tazobactam, cefoperazone-sulbactam, and amikacin. These antibiotics are currently used to combat bacterial infections. Nevertheless, this raises the question of what alternatives would be left if these organisms were found to harbour ESBL, AmpC, NDM-1, and Col-R together. We can speculate that this could lead us into the post-antibiotic era, where we would have no remaining options to treat XDR strains.

The prevalence of the *mcr-1* gene has been reported in different animals from 28 countries [29]. The *mcr* gene is more frequently isolated from animal strains than from human bacterial strains [5]. We found that 98.2% of our bacterial strains from both the animal and human sources contained *mcr-1*. Two cases (1.8%) of *mcr-2* were found, both in *P. mirabilis* isolated from animal sources, while none of the other *mcr* variants were found in our study. The presence of plasmid-mediated *mcr* resistance has been reported in different regions around the world [5, 21–23]. A study in Argentina reported 149 (49%) cases of Col-R in *E. coli* isolated from poultry, and all of them harboured the *mcr-1* gene [30]. The coexistence of *mcr-1* genes from animal, clinical, and environmental sources has also been reported in several Asian countries [31]. The *mcr-1* gene is primarily found in *E. coli* and *K. pneumoniae* of human origin, which is in line with our study. The plasmid-mediated Col-R possibly developed in animals and was ultimately transmitted to humans [5, 32, 33].

The four human isolates which harboured *mcr-1* in our study were isolated from one septic ICU patient and three patients with urinary tract infections from different wards. We did not find any history of travel or previous use of colistin for these patients. The exact source of *mcr-1* could also not be established in an Egyptian and a Polish study; however, some evidence implicated the community exposure of the patients [34, 35]. The detection of *mcr-1* and *mcr-2* in *P. mirabilis* in our study may be the first report of *mcr* genes identified in an intrinsically Col-R organism. This finding may not be significant as far as antibacterial resistance is concerned, and *mcr* genes are probably not usually searched for in an intrinsically resistant organism. However, this finding indicates the potential danger of the dissemination of *mcr*-mediated drug resistance to susceptible bacterial strains.

This study had few limitations. One limitation of our study is that we were not able to perform the broth microdilution test on all Col-R strains because of financial and time limitations. Second, we could not establish a definite route for acquiring *mcr* genes in clinical settings. Moreover, in the statistical comparison of drug resistance, having fewer bacterial isolates in one category could have affected the statistical analysis.

## Conclusions

The plasmid-mediated Col-R in GNRs among poultry is a significant emerging problem. The transfer of *mcr* genes to human bacterial strains represents a danger for patients with XDR infections. The use of colistin to promote growth in animals and increase agriculture production and its indiscriminate use in clinical settings are potential reasons for the dissemination of plasmid-mediated Col-R. We identified considerable animal reservoirs harbouring *mcr* genes that could be transferred to environmental and human strains, leading to acquired Col-R. A crucial finding of this study was the detection of the *mcr-2* gene in intrinsically Col-R *P. mirabilis*, as it could lead to the uncontrolled spread of *mcr* genes among animals and human microbiota. The rationale for the use of colistin and its availability for livestock use without a prescription should be critically reviewed to decrease the dissemination of Col-R bacteria in humans and animals.

## Data Availability

All the data supporting the findings are presented in the manuscript.

## Abbreviations

Col-R: Colistin resistance
mcr: Mobile colistin resistance
MIC: Minimum inhibitory concentration
PCR: Polymerase chain reaction
QC: Quality control
XDR: Extensively drug-resistant
ESBLs: Extended-spectrum beta-lactamases
GNR: Gram-negative rods
